# Supplementary material for: Unlocking the power of reticulocyte analysis: Advancing care for patients with abnormal haemoglobin variants at Eastern Regional Hospital, Koforidua
Source: PLoS One. 2025 May 9;20(5):e0323056. doi: 10.1371/journal.pone.0323056 (PMC12063815; doi:10.1371/journal.pone.0323056)
Supplement: Attachment — Submitted filename: Comments for 30332.docx [file pone.0323056.s002.docx]

**REVIEWER’S COMMENTS**

**Abstract**

1. The abstract could be enhanced by briefly mentioning the clinical implications of the findings, particularly how reticulocyte analysis could influence patient care.
2. A more detailed summary of limitations would be beneficial, as this would provide a realistic perspective on the study’s generalizability.

**Introduction**

The introduction clearly articulates the importance of the study, linking reticulocyte analysis to the management of abnormal hemoglobin variants, which is a valuable diagnostic gap. However, I recommend the following for improvements:

1. Key terms, such as “production defects” and “maturation defects,” are introduced but not adequately defined. Providing clear definitions upfront would enhance readability, especially for a broader audience.
2. The introduction could be strengthened by mentioning prior studies, if any, that relate reticulocyte parameters to hemoglobinopathies. This would provide a clearer context for the study's novelty.

**Methods**

1. The use of convenience sampling and the limited three-month recruitment period introduce potential biases. The methodology should address the possible impact of this sampling approach on the findings.
2. The Methods section does not mention controlling for confounding factors (e.g., nutritional status, infection history) that could affect both reticulocyte indices and hemoglobin levels. Including these factors would strengthen the study’s rigor.
3. The lack of a control group limits the ability to compare findings against a baseline. Including or discussing a control group (healthy individuals or those without abnormal hemoglobin variants) would make results more meaningful.

**Results**

1. While informative, the visuals could benefit from more descriptive labels and clearer footnotes. For example, Figure 1’s asterisks lack a corresponding explanation about their statistical significance levels.
2. The high prevalence of maturation defects (91.7%) is reported, but the clinical significance of this finding is not thoroughly discussed here. Readers would benefit from a brief comment on why this statistic is relevant for clinical settings.

**Discussion**

Links to prior studies and theories (e.g., chronic hemolytic episodes and organ damage) are well integrated, supporting the study's interpretation. The following are some areas for Improvement:

1. The discussion should acknowledge the potential influence of unmeasured factors like nutritional status or recent blood transfusions, which could impact reticulocyte counts.
2. The manuscript would benefit from a clearer emphasis on the clinical applications of fluorescence reticulocyte measurements in patient management and treatment planning.
3. Without a control group, it is challenging to contextualize the results within a standard population. The discussion should reflect on this limitation and suggest how future studies might address this gap.

**Conclusion**

1. The conclusion could benefit from more specific recommendations for clinical practice. While the potential for reticulocyte analysis is highlighted, providing explicit guidance on how these findings could alter current protocols would make the conclusion more impactful.
2. A broader reflection on the study’s limitations and suggestions for future research, especially the need for multicenter studies, would also improve the depth of this section.

**Limitations and Recommendations**

1. Additional discussion on sampling limitations (e.g., convenience sampling) and their impact on data generalizability would provide readers with a fuller perspective on the study's constraints.
2. The recommendations are valid but could be enhanced by proposing specific avenues for future research, such as exploring the role of reticulocyte indices in therapeutic monitoring for hemoglobinopathies.

**Language and Technical Presentation**

1. A few spelling errors (e.g., “recticulocytes” instead of “reticulocytes”) and minor grammatical mistakes were found, which could detract from the professionalism of the manuscript. Careful proofreading would resolve these.
2. Consistently defining and using terms throughout would improve readability, especially for an international audience not familiar with specific local practices.

**General Comments**

This manuscript presents a valuable contribution to the field of hematology, particularly in the diagnostic and management strategies for patients with abnormal hemoglobin variants in Ghana. The study’s focus on reticulocyte parameters as potential diagnostic tools is a promising approach, especially in regions where hemoglobinopathies are prevalent and resources may be limited. The application of multiple statistical methods and the clear structure of the manuscript make the study relatively easy to follow, adding to its readability.

However, a few critical aspects should be considered for improvement to enhance the manuscript’s scientific rigor and applicability:

1. The reliance on a convenience sample and the short recruitment period limits the generalizability of the findings. Additionally, the absence of a control group makes it challenging to draw definitive conclusions about the clinical significance of the reticulocyte indices compared to healthy populations. Including a control group in future studies would allow for more robust comparisons and strengthen the study’s overall validity.
2. The manuscript would benefit from a more thorough consideration of potential confounding variables, such as the nutritional status of participants, infection history, and recent transfusions, as these factors can independently influence reticulocyte indices and hemoglobin levels. Addressing or acknowledging these confounders would clarify the specific impact of reticulocyte indices in patients with abnormal hemoglobin variants.
3. While the study’s findings suggest a potential diagnostic role for reticulocyte indices, the discussion could delve further into the practical implications for patient management. Providing specific suggestions on how fluorescence reticulocyte measurements might be incorporated into existing protocols could make the findings more actionable for clinicians. Additionally, expanding on future research directions, particularly by recommending longitudinal and multi-center studies, would enhance the manuscript’s impact.
4. Minor grammatical errors and inconsistencies in terminology detract slightly from the manuscript’s professionalism. A careful proofreading to correct these issues would enhance the manuscript’s clarity and polish.
