# Supplementary material for: Unlocking the power of reticulocyte analysis: Advancing care for patients with abnormal haemoglobin variants at Eastern Regional Hospital, Koforidua
Source: PLoS One. 2025 May 9;20(5):e0323056. doi: 10.1371/journal.pone.0323056 (PMC12063815; doi:10.1371/journal.pone.0323056)
Supplement: Attachment — Submitted filename: Recommendation for the Revised Submission for 30332.docx [file pone.0323056.s004.docx]

**Recommendation for the Revised Submission**

After a careful review of the revised manuscript titled **"Unlocking the Power of Reticulocyte Analysis: Advancing care for patients with abnormal hemoglobin variants at Eastern Regional Hospital, Koforidua"**, here is my evaluation of the changes made and whether they address previous concerns adequately.

**1. General Assessment of Revisions**

The authors have made significant improvements in response to the feedback provided. They have addressed key concerns raised during the initial review, and the manuscript now presents a clearer, more concise, and scientifically rigorous study. Below is a breakdown of the major areas of improvement:

**2. Specific Responses to Reviewer Feedback**

- **Study Site Description**: The previously lengthy description of the study site has been condensed, making it more focused and relevant. This change improves the flow of the manuscript without losing any essential details.
- **Ethics and Consent**: The distinction between assent for minors and consent for adults has been clarified, reflecting ethical best practices.
- **Defining Key Terms**: The addition of precise definitions for terms like "production defects" and "maturation defects," along with references, improves readability and ensures that readers clearly understand the context.
- **Demographic Details**: While the authors opted to keep the IQR for the median age instead of a range, they addressed suggestions to revise gender descriptions, aligning with the predominantly pediatric nature of the study population.
- **Tables and Figures**: The tables now have better alignment, and figures include explanatory notes for statistical significance markers, addressing previous concerns about clarity.

**3. Major Improvements Noted**

- **Methodological Clarity**: The authors have acknowledged the limitations of their sampling method and the lack of a control group. They also provide suggestions for how these issues can be addressed in future research.
- **Clinical Implications**: The discussion now emphasizes the potential role of reticulocyte indices as diagnostic biomarkers and tools for monitoring disease progression and treatment. This addition strengthens the relevance of the study to clinical practice.
- **Acknowledgement of Limitations**: The study’s limitations, such as the absence of confounding factors like nutritional status and transfusion history, are now clearly stated, demonstrating transparency.
- **Language and Style**: Issues with spelling, grammar, and terminology, such as the misuse of "recticulocytes," have been corrected. The manuscript now reads more professionally.

**4. Remaining Concerns**

While the manuscript is much improved, a couple of areas could still benefit from minor refinements:

- **Figures vs. Tables**: While the authors prefer figures for visualizing data, adding supplementary tables to present key associations numerically could enhance interpretability for some readers.
- **Control Group**: Although the lack of a control group is acknowledged as a limitation, its absence still limits the study's ability to provide comparative insights.

**5. Final Recommendation**

The authors have clearly worked hard to address the feedback, and the revised manuscript is much stronger as a result. It offers valuable insights into reticulocyte analysis and its clinical relevance for managing patients with abnormal hemoglobin variants, particularly in resource-limited settings.

I recommend the revised manuscript as **acceptable for publication**, with only minor, optional suggestions for further enhancement, such as the inclusion of supplementary tables for additional clarity.

These findings contribute meaningfully to the field and provide a solid foundation for future research on this topic.
