# Supplementary material for: Unlocking the power of reticulocyte analysis: Advancing care for patients with abnormal haemoglobin variants at Eastern Regional Hospital, Koforidua
Source: PLoS One. 2025 May 9;20(5):e0323056. doi: 10.1371/journal.pone.0323056 (PMC12063815; doi:10.1371/journal.pone.0323056)
Supplement: Attachment — Submitted filename: Reponse to Reviewers.docx [file pone.0323056.s005.docx]

Dear editor,

We appreciate the opportunity to revise and resubmit our manuscript titled “Unlocking the Power of Reticulocyte Analysis: Advancing care for patients with abnormal haemoglobin variants at Eastern Regional Hospital, Koforidua” (Manuscript ID: PONE-D-24-30332R1). We would like to thank you and the reviewers for your insightful and constructive comments, for the second round of review which have helped us improve the quality of our work.

Below, we have provided detailed responses to editors comments as reviewers have acknowledged that all suggested comments were addressed in the previous round of review. Our current revisions have been made accordingly, and changes in the manuscript are highlighted.

### Journal Requirements

**Comment:** Please review your reference list to ensure that it is complete and correct. If you have cited papers that have been retracted, please include the rationale for doing so in the manuscript text, or remove these references and replace them with relevant current references. Any changes to the reference list should be mentioned in the rebuttal letter that accompanies your revised manuscript. If you need to cite a retracted article, indicate the article’s retracted status in the References list and also include a citation and full reference for the retraction notice.

**Response:** References were checked for completeness. No changes have been made. Thank you

**Comment**: “Overall the manuscript has merit and meets the standards for the publication but the abstract part is too long (391 words) it is hard to follow. Please shorten it. And the language with the abstract part is somehow challenging. And some parts of the abstract is written and generated by AI. Please remove the parts that was generated by AI and re-write the abstract. It didn't make sense to write "this study" when reporting your own manuscirpt. Please change or correct it.”

**Response:** The manuscript abstract has been revised manually to reflect editors suggestions and comments. See line 21-44.

Reviewers' comments:

1. If the authors have adequately addressed your comments raised in a previous round of review and you feel that this manuscript is now acceptable for publication, you may indicate that here to bypass the “Comments to the Author” section, enter your conflict-of-interest statement in the “Confidential to Editor” section, and submit your "Accept" recommendation.

Reviewer #1: All comments have been addressed

Reviewer #2: All comments have been addressed

Reviewer #3: All comments have been addressed

 2. Is the manuscript technically sound, and do the data support the conclusions?

The manuscript must describe a technically sound piece of scientific research with data that supports the conclusions. Experiments must have been conducted rigorously, with appropriate controls, replication, and sample sizes. The conclusions must be drawn appropriately based on the data presented.

Reviewer #1: Yes

Reviewer #2: Yes

Reviewer #3: Yes

 3. Has the statistical analysis been performed appropriately and rigorously?

Reviewer #1: Yes

Reviewer #2: Yes

Reviewer #3: Yes

 4. Have the authors made all data underlying the findings in their manuscript fully available?

The [PLOS Data policy](http://www.plosone.org/static/policies.action#sharing) requires authors to make all data underlying the findings described in their manuscript fully available without restriction, with rare exception (please refer to the Data Availability Statement in the manuscript PDF file). The data should be provided as part of the manuscript or its supporting information, or deposited to a public repository. For example, in addition to summary statistics, the data points behind means, medians and variance measures should be available. If there are restrictions on publicly sharing data—e.g. participant privacy or use of data from a third party—those must be specified.

Reviewer #1: Yes

Reviewer #2: Yes

Reviewer #3: Yes

 5. Is the manuscript presented in an intelligible fashion and written in standard English?

PLOS ONE does not copyedit accepted manuscripts, so the language in submitted articles must be clear, correct, and unambiguous. Any typographical or grammatical errors should be corrected at revision, so please note any specific errors here.

Reviewer #1: Yes

Reviewer #2: Yes

Reviewer #3: Yes

**6. Review Comments to the Author**

Please use the space provided to explain your answers to the questions above. You may also include additional comments for the author, including concerns about dual publication, research ethics, or publication ethics. (Please upload your review as an attachment if it exceeds 20,000 characters)

**Reviewer #1:** This is an interesting study with potential impact on clinical practice.

**Response:** Thank you for dedicating your time to reviewing our manuscript. We, the authors, greatly appreciate your expertise and insights in enhancing the quality of our work..

**Reviewer #2:** All comments reserved as authors have addressed all comments. Authors have adequately addressed my comments raised in a previous round of review.

**Response:** Thank you for dedicating your time to reviewing our manuscript. We, the authors, greatly appreciate your expertise and insights in enhancing the quality of our work.

**Reviewer #3:** Issues highlighted in the first review have been addressed by the authors. I am satisfied with the corrections implemented and the current state of the manuscript.

**Response:** Thank you for dedicating your time to reviewing our manuscript. We, the authors, greatly appreciate your expertise and insights in enhancing the quality of our work.

 7. PLOS authors have the option to publish the peer review history of their article ([what does this mean?](https://journals.plos.org/plosone/s/editorial-and-peer-review-process#loc-peer-review-history)). If published, this will include your full peer review and any attached files.

If you choose “no”, your identity will remain anonymous but your review may still be made public.

**Do you want your identity to be public for this peer review?** For information about this choice, including consent withdrawal, please see our [Privacy Policy](https://www.plos.org/privacy-policy).

Reviewer #1: No

Reviewer #2: **Yes:** Juliana Aggrey

Reviewer #3: No
